# Tissue Optical Clearing Imaging from Ex vivo toward In vivo

**DOI:** 10.34133/bmef.0058

**Published:** 2024-09-12

**Authors:** Dan Zhu, Valery Tuchin

**Affiliations:** ^1^ Britton Chance Center for Biomedical Photonics–MoE Key Laboratory for Biomedical Photonics, Advanced Biomedical Imaging Facility–Wuhan National Laboratory for Optoelectronics, Huazhong University of Science and Technology, Wuhan 430074, Hubei, China.; ^2^ Institute of Physics and Science Medical Center, Saratov State University, Saratov 410012, Russian Federation.; ^3^ Laboratory of Laser Molecular Imaging and Machine Learning, Tomsk State University, Tomsk 634050, Russian Federation.; ^4^ Institute of Precision Mechanics and Control, FRS “Saratov Scientific Centre of the RAS”, Saratov 410028, Russian Federation.

Organisms are composed of a vast number of highly specialized cells with characteristic physiological functions determined by their structure. Therefore, understanding physiological and pathological mechanisms requires obtaining 3-dimensional (3D) structural information at the cellular level across tissues, organs, and even the whole body. However, the scattering and absorption of tissue limits the penetration depth of light and thus extremely reduces the imaging performance in deep tissue. It is well known that 3D imaging outperforms 2D imaging in various scenarios for 3D reconstruction of tissue structures. This includes tracing convoluted 3D structures like vasculature, neurons, and prostate glands, as well as elucidating intricate distributions of cells or lesions. Additionally, 3D imaging excels in recognizing sparse or rare objects such as tumor progenitor cells and minimal residual disease. Traditional approaches usually involve serial tissue slicing, staining, and 2D imaging, followed by 3D reconstruction of tissue structures, which inevitably results in information loss due to slice loss or deformation, affecting its integrity. The researchers have been trying to develop advanced imaging techniques or new fluorescent probe to improve the depth of tissue optical imaging to a certain extent. In contrast, tissue optical clearing (TOC) starts from a new perspective of breaking through the limitation of turbid characteristic to tissue optical imaging, involving reducing the light scattering by homogenizing refractive indices (RIs) of various components and minimizing light absorption by removal of pigments in tissues.

In the past decade, various physical and chemical approaches have been adopted for tissue dehydration, delipidation, decolorization, and collagen dissociation and providing spatially uniform or correlated profile of the RI, with the final RI matching solution to make the tissue transparent [1]. Utilizing different clearing strategies, researchers have developed various TOC methods [2–4]. They were assigned to 2 categories based on the composition of their final RI matching solution, including solvent-based (hydrophobic) methods, such as 3DISCO, uDISCO, FDISCO, iDISCO, SHANEL, BoneClear, and PEGASOS/TESOS, and aqueous-based (hydrophilic) methods, such as Sca*l*eS, CUBIC-series, MACS, and OPTIClear, and a specific hydrophilic subtype method based on hydrogel embedding, such as CLARITY, PACT, SWITCH, and SHIELD. Originally, the hydrophobic methods were faster and shrunk tissues, while the hydrophilic methods showed better fluorescence preservation and expanded tissues slightly. At present, these 2 types of methods have seen marked advancements by overcoming many of their initial limitations. Especially, their combined action can offer distinct advantages, with the HYBRiD technique serving as an exemplary model of such a synergistic integration.

TOC combined with various fluorescence labeling and light sheet fluorescence microscopy (LSFM) can achieve high-throughput, high-resolution 3D structural imaging of integrate tissues, organs, and even the whole bodies, which make it one of the most popular tools in neuroscience research. The applications of TOC technique in brain imaging were selected as the “Top Ten Breakthrough Technologies of 2013” by the journal *Science*. Professor Jeff W. Lichtman predicted in his review that TOC opens a door to new discovery of the inner workings of organisms with a view of space [4]. As he said, in the past decade, TOC technique has been widely applied in many fields of biomedical science [5–7]. More and more researchers discovered new neural circuits by imaging the transparent brain and other whole organs, such as the appetite-promoting neural network related to diet in the human hippocampus, the connection pathway between intestinal/gastric sensory nerves and brain neurons, and the neural–fat connection pathway and regulation. TOC imaging is used to draw high-resolution 3D maps of peripheral nerves, muscles, blood vessels, and other structures and to evaluate the occurrence, development, and metastasis of tumors, as well as the combination efficiency and accuracy of targeted drugs, providing technical support for cancer treatment and targeted drug research and development. More importantly, TOC imaging not only is limited to the basic research of frontier life science but also is expected to innovate the new paradigm of clinical pathological diagnosis, extending the existing 2D pathological detection to 3D pathology. One of the biggest advantages of introducing TOCs in routine diagnosis could be the speeding; the other could be the slicing procedure skipping, which is often error prone. The accurate diagnosis will provide patients with better treatment options. While TOC technique has been utilized to visualize various human tissues such as brain blocks, embryos, and prostates, its integration into routine diagnosis presents substantial challenges. In addition to the well-known regulatory issues associated with new clinical technology, there are significant technical hurdles to be addressed. The diverse nature of clinical tissue samples and pre-analytic factors, including sample pre-processing and storage, imposes stringent demands on clearing methods. Simultaneous advancements in high-efficiency whole-mount tissue labeling, image processing, and analytical techniques are essential. The tissue clearing workflow involves complex reagent preparation and multiple steps. Quality sample preparation relies heavily on the expertise of technicians, yet clinical samples cannot afford any risk of failure, necessitating standardized processing for clearing. However, the majority of current clearing protocols still hinge on the manual operations that are labor intensive, hence inefficient to handle the extensive volume of clinical samples, mandating the introduction of automatic equipment to meet these demands. X-CLARITY, as a commercial equipment for the renowned CLARITY clearing technique, has set a commendable benchmark for instrumentation. Moreover, the adoption of TOC in diagnosis requires compelling evidence of its clinical utility, making it a crucial consideration. Overcoming these challenges holds the promise of leveraging TOC for assisting in the construction of human health or disease information resource libraries in the future.

The successful applications of TOC imaging profit from the development of various labeling and LSFM. The key to perform complete and precise labeling of desired biological components before clearing [7], such as nucleic acids, neural circuits, vasculature, or specific proteins with transgenic labeling, molecular labeling, or immunolabeling methods is well known. However, there are still challenges, i.e., the limited penetration and diffusion of immunolabeling fluorescent labels into deep tissues can result in uneven staining and incomplete visualization of structures, particularly in larger samples; nonspecific binding and background fluorescence can obscure the signal of interest, especially in complex tissue samples; the compatibility of fluorescent labels with various tissue clearing agents is also not always well established, which can affect the efficiency of the clearing process and the preservation of tissue structures. LSFM illuminates the vicinity of the focal plane and rapidly captures 3D images, significantly reducing illumination dose while achieving higher temporal resolution, which have almost become common equipment for universities or institutions [8]. In recent years, various LSFM modalities have been developed, from dual-objective to single-objective setup to enhance the imaging speed, and epi-illumination selective plane illumination microscopy (SPIM) and high-numerical aperture (NA) glass-tipped remote focusing objectives, successfully enhancing resolution and fluorescence detection efficiency, making single-objective LSFM as convenient as confocal microscopy while retaining superior speed and low phototoxicity. Compared with the constantly emerging commercial or laboratory-built LSFM, there are few tissue clearing instruments. The complex clearing treatment processes still rely on manual implementation, and the manpower and material resources consumed will be unimaginable as demand increases. Thus, exploiting tissue clearing instruments to replace manual implementation of clearing sample preparation should be the imperative way.

Besides the ex vivo TOC imaging, the concept and corresponding techniques of switchable and chronic optical windows were developed to achieve in vivo vascular and neural imaging with high resolution and provide effective ultraviolet (UV), photodynamic, and photothermal therapies [1,9–16]. Generally, researches had to establish dorsal skin chamber and cranial skull window, but surgical operation inevitably causes side effects. In vivo optical clearing switchable windows enable image dermal or cortical optical structure and function, and image the spectral range from UV to visible and further to near infrared. Recently, a through-intact-skull chronic window was developed for cortical structure and function observation in mice. It almost overcomes limitations of other skull windows and enables the observation of an immune response on a bilateral cortical scale at single-cell resolution after traumatic brain injury without affecting the pathological environment of the brain. This window also has the advantages of craniotomy freeness, centimeter field of view, synaptic resolution, large imaging depth, long-term observation capability, and suitability for awake mice. In addition, through this skull window, the photodynamic effect was applied to open the blood–brain barrier or induced targeted embolization of cortical vessels, which provides a potential tool for stroke mechanisms or drug effect evaluation. It holds great potential for physiological and pathological research in brain science and many other biomedical fields.

In vivo TOC has also been applied to human subjects, such as the use of aqueous solutions of glucose, sucrose, and fructose, as well as polyethylene glycol-400 (PEG-400) on forearm and palm skin to improve the imaging quality of optical coherence tomography (OCT) [1,17]. The clinical applications of TOC methods are currently limited due to the thicker and more robust nature of human skin, particularly the stronger barrier function in the stratum corneum. Although efficient in vivo skin optical windows have been successfully established in experimental animal models, they are less effective in human skin. Therefore, the development of high-efficiency and safe in vivo TOC methods specifically designed for human skin is of great significance. In particular, clinically approved chemical skin permeability enhancers such as propylene glycol, oleic acid, and dimethyl sulfoxide, as well as physical permeation methods such as dermabrasion and sonophoresis, recently have been successfully used to improve the contrast and intensity of 3D images of linear-field confocal OCT at skin depth from 70 to 400 μm after 10 min of exposure to PEG-400/oleic acid/propylene glycol mixtures in combination with dermabrasion and sonophoresis [17]. Furthermore, considering variations in human age, skin color, and body parts, it is important to establish niche-targeting skin TOC methods for specific clinical applications, such as pediatric injection, dermoscopy, and ultrasonography. These advancements will broaden the potential clinical utility of TOC techniques.

TOC technique prospective for in vivo applications in humans is working in extremely broad wavelength range from deep UV to terahertz (THz) waves with the possibility to be combined with clinically used imaging techniques like ultrasound (US), x-ray computed tomography (CT), and magnetic resonance imaging (MRI) [1,11–16]. The multimodality through the use of various combinations of optical and other imaging techniques is a challenging problem that promises many benefits due to mutually improved spatial and temporal resolution as well as the depth of imaging. Numerous optical methods demonstrate significant improvement due to TOC, namely, multiphoton imaging, OCT along with its many modifications, laser speckle contrast and polarization-sensitive imaging, confocal microscopy, functional near-infrared spectroscopy, fluorescence imaging, photoacoustic tomography, diffusion optical tomography, as well as the Raman-based techniques, including confocal Raman microscopy, surface-enhanced Raman scattering, coherent anti-Stokes Raman scattering, and stimulated Raman scattering [1].

It is important to note that optical clearing agents (OCAs) are good markers for monitoring of diabetes mellitus complications [15] and discrimination of tissue malignancy [16] through their impact on blood and lymph flow [1,15] and specificity of diffusion in living tissues, which also gives important information for optimal organ cryopreservation, as the most OCAs are cryoconservation substances [1,12].

**Fig. 1.**
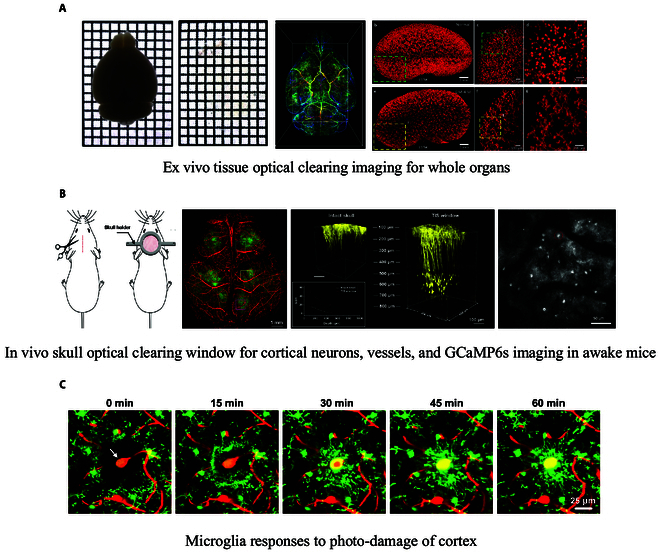
Tissue optical clearing imaging for ex vivo whole organs and in vivo cortex. (A) Ex vivo tissue optical clearing imaging for whole organs. (B) In vivo skull optical clearing window for cortical neurons, vessels, and GCaMP6s imaging in awake mice. (C) Microglia responses to photo-damaged cortex.

In conclusion, TOC is beneficial for enhancing multimodal optical imaging from ex vivo toward in vivo, also enhancing treatment efficacy. It promises that advanced biophotonic techniques working in a wide wavelength range from deep-UV to THz range can be significantly improved. The combination of optical techniques with US, CT, and MRI is possible due to usage of the unique properties of US coupling gel, and CT and MRI contrasting agents, which are also effective OCAs (Fig. 1) [2,10,18,19].
